# Analysis of the Errors Caused by Disturbed Multimode Fibers in the Interferometer with Fiber-Coupled Delivery

**DOI:** 10.3390/s20051513

**Published:** 2020-03-09

**Authors:** Yizhou Xia, Ming Zhang, Yu Zhu, Weinan Ye, Fuzhong Yang, Leijie Wang

**Affiliations:** 1 State Key Laboratory of Tribology, Department of Mechanical Engineering, Tsinghua University, Beijing 100084, China; xyz18@mails.tsinghua.edu.cn (Y.X.); zhuyu@tsinghua.edu.cn (Y.Z.); yewn12@mails.tsinghua.edu.cn (W.Y.); yfz17@mails.tsinghua.edu.cn (F.Y.); wang-lj66@mail.tsinghua.edu.cn (L.W.); 2 Beijing Lab of Precision/Ultra-Precision Manufacture Equipment and Control, Tsinghua University, Beijing 100084, China

**Keywords:** interferometer, multimode fiber, disturbance error, fringe contrast, interference angle

## Abstract

In this paper, the errors of the displacement measurement interferometer with multi-mode fiber-coupled delivery are analyzed when the fibers are disturbed. Simulation results show that the characteristic frequency of the measurement error is consistent with that of disturbance, and the error has higher order frequency components. The experiments are designed for the effect of fringe contrast on the measurement error. The experimental results show that the measurement error is rather sensitive to the interference angle between the measurement arm and the reference arm in the multi-mode fibers, but not to the irradiance ratio of the measurement arm and the reference arm. In an interferometer with multimode fiber, the interference angle between the measurement arm and the reference arm needs to be restricted. This conclusion provides a theoretical basis for designing an interferometer measurement system with interference angle that is adaptive to wider application.

## 1. Introduction

In order to achieve a higher measurement accuracy and wider application, optical fiber is used for the delivery of beams in the displacement measurement interferometer [1,2]. In the interferometer system with polarization-maintaining fibers (PMFs), the PMFs are used for the delivery of single-frequency polarized beams, the purpose of which is to isolate the laser source from the interferometer, so as to prevent the error caused by laser heating. Moreover, PMFs-coupled delivery can realize the spatial separation of measurement beam and reference beam, effectively eliminating periodic nonlinearity (PNL) [3]. Even if the error caused by perturbation of the single-mode fiber is large, it can be compensated for by means of biaxial measurement or a compensation axis [4]. There are also displacement measurement interferometer systems with PMFs and multimode fibers (MMFs), in which the MMFs are used to deliver the measurement arm and the reference arm for the purpose of separating the board card from the interferometer and reducing the noise [5,6]. Therefore, the displacement measurement interferometer with the fibers will have a smaller size and a wider range of applications [7].

There have been many advances in the research of MMFs and the sensor based on MMFs [8,9,10,11]. The theory of how the beam propagates in MMFs has also been improved. The beams propagate in MMFs with multiple modes, each of which has an effective refractive index (ERI) [12,13,14,15]. Similar to PMFs, MMFs will generate stress and deformation when disturbed. Due to the optic-stress effects, the refractive index of the fiber will change [16,17,18]. There are many interferometers, and the measurement arms and the reference arms of these interferometers cannot be guaranteed to be parallel and coincident in use [19,20,21,22,23,24]. In some applications of interferometers with MMFs such as the relative displacement measurement of the dual wafer stage, the interferometers inevitably move with one stage [25]. In this case, MMFs are disturbed by the motion of the interferometers. The measurement errors caused by disturbed MMFs needs to be studied to achieve higher measurement accuracy, which is beneficial for designing an interferometer measurement system with an interference angle that can be adapted to wider application.

In this paper, for the displacement measurement interferometer with multi-mode fiber-coupled delivery, we analyze the errors caused by disturbed MMFs and obtain the analytical expression of it. Then, simulation models are established for the solution of the errors. The variation of the ERI of the MMF under disturbance is obtained in the COMSOL simulation, and the measurement error is obtained in the ZEMAX simulation. It is verified that the characteristic frequency of the measurement error is consistent with the frequency of the disturbance. Finally, experiments are designed to verify the simulation results, and the effect of fringe contrast on the errors caused by disturbed MMFs is further studied. Fringe contrast can be express as the ratio of AC signal to DC signal (*I_AC_/I_DC_*), where the AC signal is *I_AC_* and the DC signal is *I_DC_* [26]. It is concluded that the irradiance ratio of the measurement arm and the reference arm has less influence on the errors, while the interference angle between the measurement arm and the reference arm has great influence on the errors. The larger the interference angle, the larger the errors, and the greater the rate of change of the measurement errors with *I_AC_/I_DC_*.

## 2. Principle

### 2.1. Errors Caused by Disturbed MMFs

**Figure 1:**
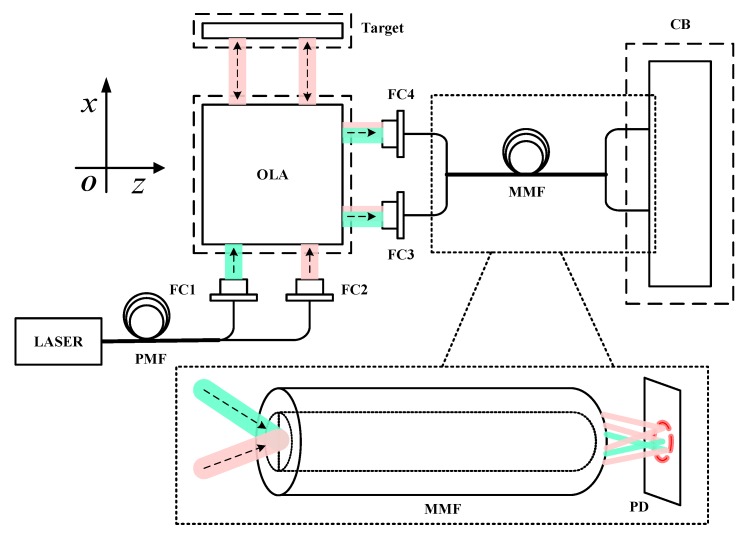
The schematic diagram of the interferometer measurement system with fiber-coupled delivery. The red beams are the measurement arms and the green beams are the reference arms.

As shown in Figure 1, in the interferometer measurement system with fiber-coupled delivery, the single-frequency polarized beams generated by the laser are delivered to the fiber couplers (FCs) by the PMFs, and then into the optical lens assembly (OLA) to form the reference arms and the measurement arms. The measurement arms carry the Doppler phase shift of the target. The reference arms and the measurement arms are coupled to the MMFs by FCs after passing through the OLA, and are finally delivered to the control board (CB), where photoelectric conversion and phase discrimination are completed. The irradiance signal received by the photodetector (PD) in the CB is the sum of each mode component of the measurement arm and reference arm. 

Deformation and strain occur in the core and cladding of MMFs when disturbed. The refractive index of the core and cladding of the MMF changes due to the optic-stress effects when the stress exists are inconsistent in the fiber, including the birefringence effects. Moreover, when the MMF deforms, the propagation path of the internal beams will change. These two points cause the ERI of each mode of the MMF to change.

We analyzed the errors in the case of heterodyne interferometer. In MMFs, the electric field of the measurement arm (*E_m,j_*) and the electric field of the reference arm (*E_r,j_*) can be expressed as:

(1) $$\begin{array}{l} E_{m,j}=E_{1}e^{2\pi f_{1}t+\psi +\Phi_{1}+\frac{2\pi }{\lambda }Ln_{eff,m,j}}\\ E_{r,j}=E_{2}e^{2\pi f_{2}t+\Phi_{2}+\frac{2\pi }{\lambda }Ln_{eff,r,j}} \end{array}$$

where *j* is the modulus, *n_eff,m,j_* is the *j*-th ERI of the measurement arm, *n_eff,r,j_* is the *j*-th ERI of the reference arm, *L* is the length of the MMF, *λ* is the vacuum wavelength of the laser, *f*_1_ and Φ_1_ are the frequency and initial phase of the measurement arm, respectively, *f*_2_ and Φ_2_ is frequency and the initial phase of the reference arm, respectively, and *ψ* is the Doppler-phase-shift of the target.

The effect of disturbance on the signals can be equivalent to the effect on the ERI of the MMF. The irradiance signals detected by PD is of the form:

(2) $$\begin{array}{ll} I= & \sum E_{m,j}^{2}+\sum E_{r,j}^{2}+\sum_{j\neq i}2\left|E_{r,j}\right|\left|E_{r,i}\right|\cos(\Delta \beta_{r,j,i})+\sum_{j\neq i}2\left|E_{m,j}\right|\left|E_{m,i}\right|\cos(\Delta \beta_{m,,j,i})\\ & +\sum \sum 2\left|E_{m,j}\right|\left|E_{r,i}\right|\cos(2\pi (f_{1}-f_{2})t+\psi +(\Phi_{1}-\Phi_{2})+\Delta \varphi_{j,i}) \end{array}$$

where Δ*β_r,j,i_*, Δ*β_m,j,i_*, and Δ*φ_j,i_* can be expressed as follows:

(3) $$\begin{array}{l} \Delta \beta_{r,j,i}=\frac{2\pi }{\lambda }L(n_{eff,r,j}-n_{eff,r,i})\\ \Delta \beta_{m,j,i}=\frac{2\pi }{\lambda }L(n_{eff,m,j}-n_{eff,m,i})\\ \Delta \varphi_{j,i}=\frac{2\pi }{\lambda }L(n_{eff,m,j}-n_{eff,r,i})=\frac{2\pi }{\lambda }L\Delta n_{eff,j,i} \end{array}$$

Because phase discrimination is mainly aimed at the high frequency part of Equation (2), Δ*β_r,j,i_* and Δ*β_m,j,i_* are filtered by a high-pass filter. The filtered signal can be expressed as:

(4) $$I_{f}=\sum \sum 2\left|E_{m,j}\right|\left|E_{r,i}\right|\cos(2\pi (f_{1}-f_{2})t+\phi +(\Phi_{1}-\Phi_{2})+\Delta \varphi_{j,i}).$$

It can be deduced from Equations (3) and (4) that the errors caused by disturbed MMFs can be expressed as,

(5) $$\alpha =\arctan\frac{\sum \sum \sin(\frac{2\pi }{\lambda }L\Delta n_{eff,j,i})}{\sum \sum \cos(\frac{2\pi }{\lambda }L\Delta n_{eff,j,i})}.$$

According to Equation (5), α varies with Δ*n_eff,j,i_*. However, Δ*n_eff,j,i_* is mainly affected by two factors: the refractive index of the MMF and the path of the beams. As for the influence emerging from the refractive index of the MMF, when stress is generated inside the MMF, based on optic-stress effects, its refractive index can be expressed as [17,18],

(6) $$\left[\begin{array}{c} n_{x}(t)\\ n_{y}(t)\\ n_{z}(t) \end{array}\right]=\left[\begin{array}{c} n_{0}\\ n_{0}\\ n_{0} \end{array}\right]-\left[\begin{array}{ccc} B_{1} & B_{2} & B_{2}\\ B_{2} & B_{1} & B_{2}\\ B_{2} & B_{2} & B_{1} \end{array}\right]\left[\begin{array}{c} S_{x}(t)\\ S_{y}(t)\\ S_{z}(t) \end{array}\right],$$

where *n_x_*, *n_y_*, and *n_z_* are the diagonal elements of the refractive index tensor, *n*_0_ is the refractive index of the amount of material in the stress-free state, *B*_1_ and *B*_2_ are optic-stress constants which dependent on the material, and *S_x_*, *S_y_*, and *S_z_* are the diagonal elements of the stress tensor. It should be noted that the refractive index of the fiber material here adopts a diagonal model, ignoring the off-diagonal elements of the refractive index tensor.

Through Equation (6), the refractive index *n*(*t,x,y,z*) of the MMF at different positions can be obtained. In the MMF, the path of the beams is *p*(*x,y,z*). The ERI is the integral of the refractive index gradient along *p*, so the ERI can be expressed as [13]:

(7) $$n_{eff}(t)=\frac{1}{L}\int_{p\left(x,y,z\right)}n(t,x,y,z)ds.$$

When the MMF is disturbed, not only does the internal stress affect the ERI, but also the path *P* changes. So Δ*n_eff_* in Equation (3) can be expressed as,

(8) $$\Delta n_{eff}(t)=\frac{1}{L}(\int_{p_{m}(t,x,y,z)}n(t,x,y,z)ds-\int_{p_{r}(t,x,y,z)}n(t,x,y,z)ds)$$

By substituting Equation (8) into Equation (5), the errors caused by disturbed MMFs varying with time can be obtained:

(9) $$\alpha (t)=\arctan\frac{\sum \sum \sin(\frac{2\pi }{\lambda }(\int_{p_{m}(t,x,y,z)}n(t,x,y,z)ds-\int_{p_{r}(t,x,y,z)}n(t,x,y,z)ds))}{\sum \sum \cos(\frac{2\pi }{\lambda }(\int_{p_{m}(t,x,y,z)}n(t,x,y,z)ds-\int_{p_{r}(t,x,y,z)}n(t,x,y,z)ds))}.$$

In the frequency domain, when the frequency of the disturbance on MMFs is *f*, the stress tensor and refractive index tensor in Equation (6) have the characteristic frequency *f*, and the path of the beams in MMFs also has the characteristic frequency *f*. Therefore, the errors caused by disturbed MMFs include the frequency component *f*, as well. By obtaining their mean, the spectrum of experimental results can be used to analyze the influence of different conditions on the error. 

In Equation (9), if the interference angle between the measurement arm and the reference arm is zero, then α(*t*) is also zero and there are no errors caused by disturbed MMFs in the system. If interference angle exists, then *p_m_*(*t,x,y,z*) is different from *p_r_*(*t,x,y,z*), which results in the error. The interference angle between the measurement arm and the reference arm should be within the allowable range to ensure good quality of interference.

Equation (9) can calculate the sum of the error of each mode. Although Equation (9) gives an analytical model of the error caused by disturbed MMFs, it is difficult to calculate the error only by using this formula because the specific stress distribution of the MMF and propagation path of the beams are difficult to determine. The following simulation combines COMSOL and ZEMAX to solve the error and to study the characteristics of the error.

### 2.2. Simulations

Through simulations, we verified that the disturbance of MMFs can introduce measurement error. We also determined that the characteristic frequency of the errors obtained by simulations is consistent with the frequency of disturbance. The simulations are divided into two parts. At first, the ERI of the MMF is obtained by COMSOL simulation, and then the results are substituted into the model in ZEMAX simulation to obtain the measurement error after signal processing.

In the COMSOL model, the simulation is divided into two steps. Firstly, the stress distribution and deformation distribution of the MMF under periodic load are calculated by using the interface of ‘solid mechanics’. Secondly, the obtained stress distribution is substituted into Equation (6) to calculate the refractive index of each part of the fiber after strain. The ERI of the fiber is calculated by using the ‘mode analysis’ module in the ‘wave optics’ interface.

The specification of the MMF is referred to the product of THROLABS, and the parameters in the simulations are shown in Table 1. Considering the size of the simulation grid and the amount of calculation, the length of the MMF is 1 mm. The outer diameter of the cladding of the MMF is 425 μm, and the diameter of the core is 400 μm. The Young’s modulus, Poisson’s ratio, density, and stress optical coefficients of the fiber refer to the parameters of silica.

**Figure 2:**
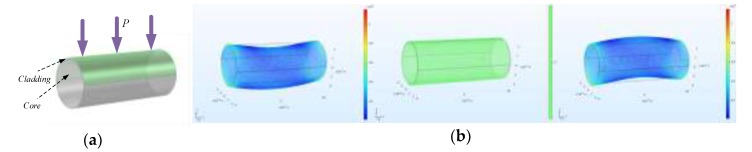

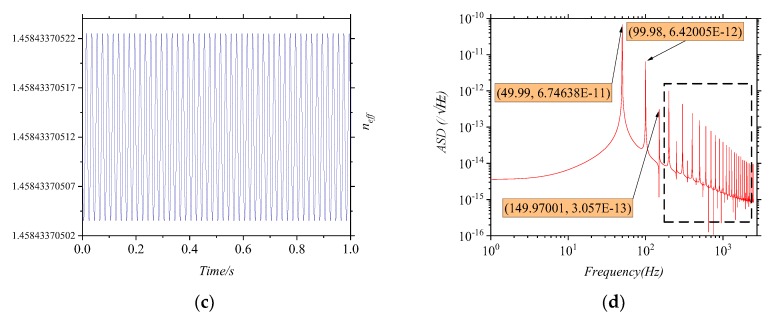
(**a**) The COMSOL model of multimode fibers (MMFs) with load P applied to the upper surface of the cladding; (**b**) The figures of the MMF’s deformation and stress distributions under the load at *t* = 2 ms, *t* = 10 ms, and *t* = 12 ms; (**c**) The graph of the effective refractive index (ERI) of the fundamental mode; (**d**) The graph of the amplitude spectral density of the ERI.

Disturbance on the MMF is shown in Figure 2a. The applied load is the uniform pressure *P*, and its expression is *P* = 100sin(2*πf_L_t*) Pa. The frequency of the load is *f_L_* = 50 Hz. The sampling time is 1 second and the sampling frequency is 5 kHz. Figure 2b shows the deformation and stress distribution of the fiber at *t* = 2 ms, *t* = 10 ms, and *t* = 12 ms, respectively. The stress distribution is substituted into Equation (6) to calculate the refractive index distribution, and then the ERI is obtained by mode analysis. In order to study the characteristics of the error in the frequency domain, we mainly focused on the ERI of the fundamental mode because the error in the fundamental mode is larger than that in the higher-order mode. The ERI of the fundamental mode is shown in Figure 2c. For the sake of getting a more intuitive conclusion, the amplitude spectral density (ASD) of the ERI was analyzed, which is shown in Figure 2d. It can be seen that the first order frequency of the ERI is 50 Hz, which is consistent with the frequency of the applied load. Meanwhile, in the black box in Figure 2d, the ERI also has the higher-order frequencies, which are multiples of the first-order frequency.

**Figure 3:**
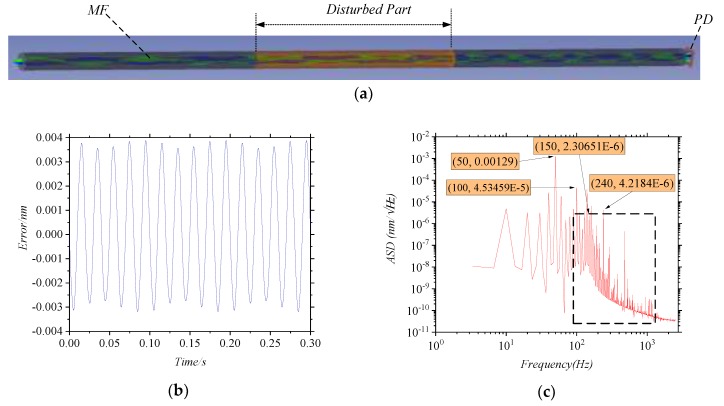
(**a**) The ZEMAX model of MMF; In (**a**), The disturbed part is the middle section of optical fiber. (**b**) the graph of the measurement error and time when the MMF is disturbed; (**c**) the graph of the amplitude spectral density of the measurement error.

Since the change of the ERI is small and the actual length of the MMF is much longer than 1 mm in the COMSOL model, we first amplify the refractive index change by using a certain factor, and then assign the values to the optical fiber in ZEMAX simulation. As shown in Figure 3a, the measurement arm and the reference arm are coupled to the MMF and finally reach the detector for processing. In this simulation, we divide the fiber into three parts, and the middle part is disturbed. The ERI obtained from COMSOL simulation is given to the MMF in the disturbed part, and the final error is obtained by calculating the irradiance signals detected by PD. In the ZEMAX model, the frequency difference between the measurement arm and the reference arm is 240 Hz, the sampling frequency is 5 kHz, and the sampling time is 1 s. Figure 3b is the measurement errors from 0.35 s to 0.65 s. The ASD of the measurement errors is analyzed in Figure 3c. It can be seen that the main frequency is 50 Hz, at which the amplitude of the error is 0.00129 nm/√Hz. There is a second-order frequency of 100 Hz, which is consistent with the ERI’s and the load’s frequencies. In the black box in Figure 3c, the ASD of the error has the higher-order frequencies as well. The component at 240 Hz is the error of phase discrimination process, which is consistent with the frequency difference.

Using theoretical analysis and simulations results, it was verified that when the MMF is disturbed, the ERI change with time, which results in the measurement error. The frequency of the ERI and the frequency of the error caused by disturbed MMFs are consistent with the frequency of the disturbance, and there are the octave components of the disturbance.

## 3. Experiments

### 3.1. Experimental Principle

**Figure 4:**
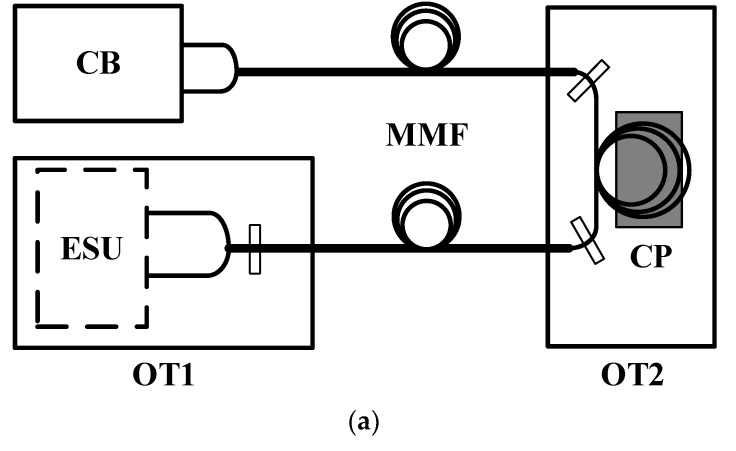

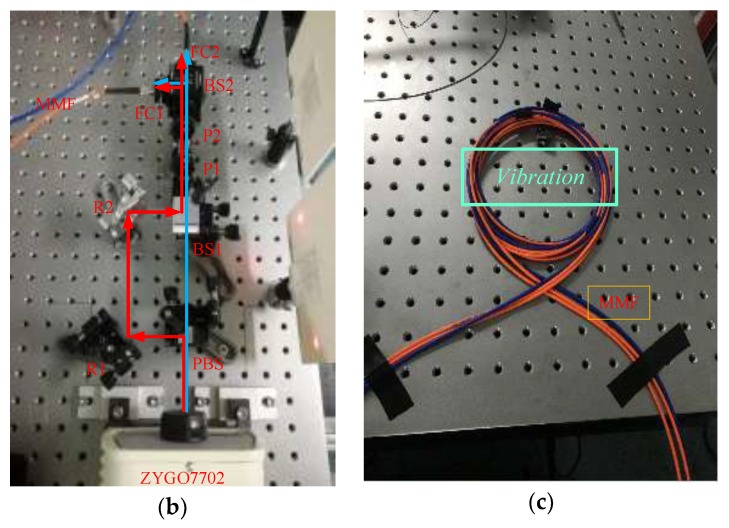
(**a**) The schematic diagram of the experiments. (**b**) The schematic diagram of experimental set-up on OT1. PBS is a polarizing beamsplitter, BS1 and BS2 are beamsplitters, P1 and P2 are polarizing plates, R1 and R2 are reflectors, and FC1 and FC2 are fiber couplers. (**c**) the MMFs are fixed on OT2 and disturbed by the cellphone.

To explore the characteristics of the error caused by disturbed MMFs, the experiment is shown in Figure 4a. To prevent experimental set-up (ESU) from being affected by the vibration, ESU was installed on the first optical stage (OT1), and a segment of MMF was fixed on the second optical stage (OT2). In Figure 4b, the laser (model: ZMI7702 Laser Head, ZYGO, Inc, Middlefield, CT, USA) is the dual frequency laser which emits two beams that are linearly polarized in a coaxial direction. They have different frequencies and have orthogonal polarization states. After the dual-frequency beams passes through polarizing beamsplitter (PBS), it is divided into p-polarized beams and s-polarized beams, and then takes a different death path to form the measurement arm and the reference arm. After passing beamsplitter (BS1) and polarizing plate (P1), the measurement arm interferes with the reference arm. The interference beams are divided into two beams through beamsplitter (BS2), and then pass through fiber couplers (FCs) into MMFs, which are finally delivered to the control board (CB) (model: ZMI4100TM Series Measurement Board, ZYGO, Inc, Middlefield, CT, USA) for processing. As shown in Figure 4c, a segment of MMF is fixed on OT2, on which a cellphone is placed to vibrate at a fixed frequency and amplitude.

Air disturbance and temperature drift have a great influence on the measurement in the laser interferometer. In order to eliminate the influence of these errors and obtain the error caused by disturbed MMFs, the interference beams through BS2 are divided into two beams. The beams are delivered by different MMF. Two measurement signals *I*_1_ and *I*_2_ can be expressed as:

(10) $$\begin{array}{l} I_{1}\propto \cos(2\pi \Delta ft+\varphi_{T}+\varphi_{D}+\varphi_{MMF1})\\ I_{2}\propto \cos(2\pi \Delta ft+\varphi_{T}+\varphi_{D}+\varphi_{MMF2}) \end{array}$$

where *φ_T_* is the temperature drift error, *φ_D_* is the dead range error, and *φ_MMF_*_1_ and *φ_MMF_*_2_ are the errors caused by MMFs. The difference of the measurement signals only related to the error caused by disturbed MMFs can be expressed as:

(11) $$\begin{array}{l} Error_{1}=\frac{\varphi_{T}+\varphi_{D}+\varphi_{MF1}}{2\pi }\lambda \\ Error_{2}=\frac{\varphi_{T}+\varphi_{D}+\varphi_{MF2}}{2\pi }\lambda \\ DE=Error_{1}-Error_{2}=\frac{\varphi_{MF1}-\varphi_{MF2}}{2\pi }\lambda \end{array}.$$

### 3.2. Results and Analysis

By comparing the difference between *Error*_1_ and *Error*_2_ (*DE*) before and after the disturbance, the influence of the disturbance on the signals can be obtained. Measurement data in 30 s is recorded in one experiment. The MMF is stationary for the first 10 s and disturbed by for the last 10 s.

**Figure 5:**
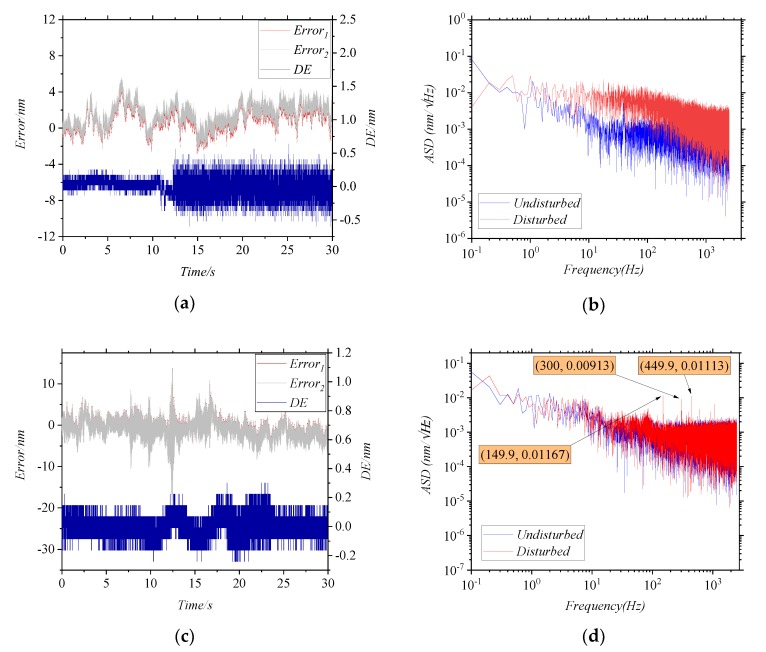
(**a**,**c**) show that *Error*_1_ and *Error*_2_ vary with time, and the blue line is the difference value *DE*. (**b**,**d**) show the ASD of the *DE* in (**a**,**c**), respectively. (**a**,**b**) are the results of the disturbance by the hands. (**c**,**d**) are the results of the disturbance by the cellphone.

In Figure 5a,b, the MMFs are disturbed by the hands. The results of *DE* in the first 10 s and in the last 10 s are significantly different, which indicates that the error increases significantly when the MMFs are disturbed. To get the influence factors of error more accurately, the results of cellphone disturbance are investigated later. In Figure 5c, the MMFs are disturbed by the cellphone, and the frequency of the disturbance is 150 Hz. As shown in Figure 5d, the ASD of the *DE* before and after the disturbance is different, and it can be seen that these characteristic frequencies appear after the disturbance: 149.9 Hz, 300 Hz, 449.9 Hz, etc. These frequencies coincide with the first-order, second-order and third-order vibration frequencies, which is consistent with the results of simulation. Meanwhile, it was verified that the disturbance of MMFs causes measurement error, and the characteristic frequency of the error matches the frequency of disturbance.

Fringe contrast is a measure of the interference quality, and a good fringe contrast is needed to accurately get measurement results. Fringe contrast not only influences the signal-to-noise ratio, but relates to the error caused by disturbed MMFs. Two factors affect *I_AC_/I_DC_*: the interference angle and the irradiance ratio. The interference angle is the angle between the vector direction of the reference arm and the vector direction of the measurement arm. The irradiance ratio is the ratio of irradiance of the reference arm to irradiance of the measurement arm. The larger the interference angle, the worse the fringe contrast. The greater the difference between the irradiance of the reference arm and the irradiance of the measurement arm, the worse the fringe contrast [26]. The influence of the factors on the errors is further studied below.

**Figure 6:**
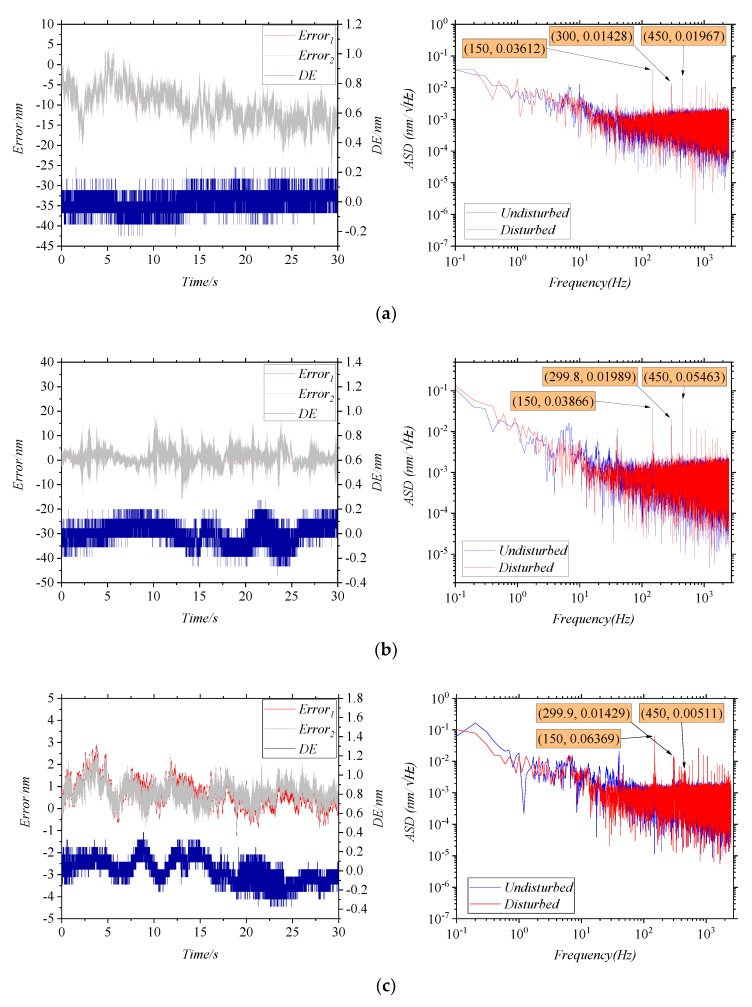

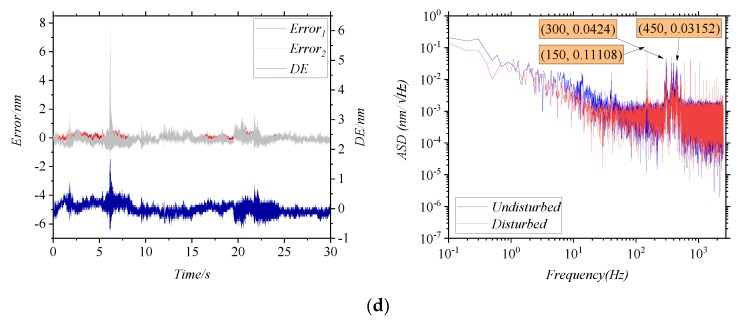
The errors curves for changing the interference angle, (**a**) *I_AC_/I_DC_* = 100%, (**b**) *I_AC_/I_DC_* = 77%, (**c**) *I_AC_/I_DC_* = 62%, (**d**) *I_AC_/I_DC_* = 53%.

The influence of the interference angle on the error caused by disturbed MMFs is studied by adjusting R1 or R2 in Figure 4b. The larger the interference angle, the smaller *I_AC_/I_DC_*. In the experiment, the frequency and the amplitude of the vibration are kept unchanged, and only the angles of the mirrors are changed. Four sets of *I_AC_/I_DC_* are determined, and then multiple sets of data are measured repeatedly under each set of *I_AC_/I_DC_*. As shown in Figure 6, the results are measured for *I_AC_/I_DC_* at 100%, 77%, 62%, and 53%, respectively. In the time domain, the 3σ of the *DE*s in four conditions are 0.1719 nm, 0.2279 nm, 0.372 nm, and 0.5115 nm, respectively. It can be seen that the worse *I_AC_/I_DC_*, the larger the 3σ of the *DE*. In the frequency domain, the ASD of the *DE*s at the first-order characteristic frequency are 0.03612 nm/√Hz, 0.03866 nm/√Hz, 0.06369 nm/√Hz, and 0.11108 nm/√Hz, respectively. It can be seen that the worse *I_AC_/I_DC_*, the larger the ASD of the *DE*.

**Figure 7:**
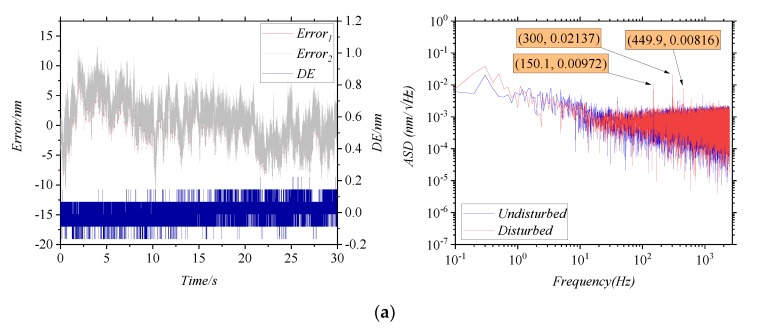

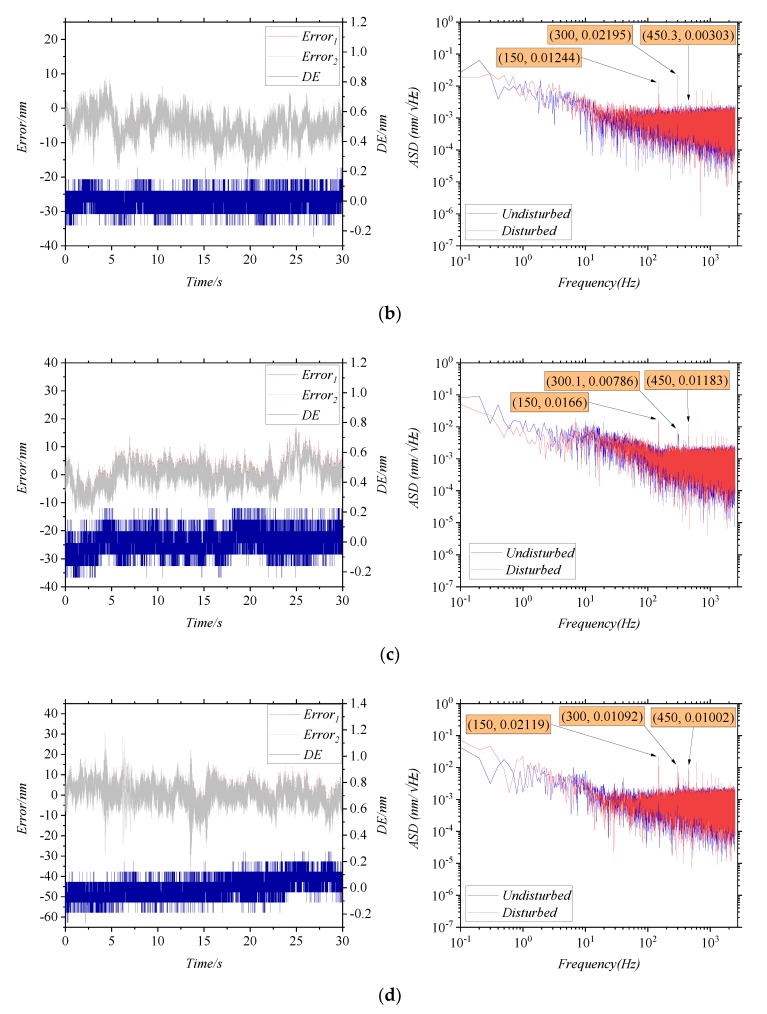
The errors curves for changing the irradiance ratio, (**a**) *I_AC_/I_DC_* = 100%, (**b**) *I_AC_/I_DC_* = 84%, (**c**) *I_AC_/I_DC_* = 77%, (**d**) *I_AC_/I_DC_* = 54%.

The influence of the irradiance ratio is studied mainly by adjusting P1 in Figure 4b. It needs to be guaranteed that the frequency and the amplitude of the vibration are kept unchanged as well. After each change in P1’s angle, *I_AC_/I_DC_* is measured. Four sets of *I_AC_/I_DC_* are determined, and then multiple sets of data are measured repeatedly under each set of *I_AC_/I_DC_*. As shown in Figure 7, the results were measured for *I_AC_/I_DC_* at 100%, 84%, 77%, and 54%, respectively. In the time domain, the 3σ of the *DE* in four conditions are 0.1518 nm, 0.1539 nm, 0.166 nm and 0.2039 nm, respectively. In the frequency domain, the ASD of the *DE*s at the first-order characteristic frequency are 0.00972 nm/√Hz, 0.01244 nm/√Hz, 0.0166 nm/√Hz and 0.02119 nm/√Hz, respectively. It can be seen that *I_AC_/I_DC_* has little effect on the 3σ and the ASD of the *DE*.

**Figure 8:**
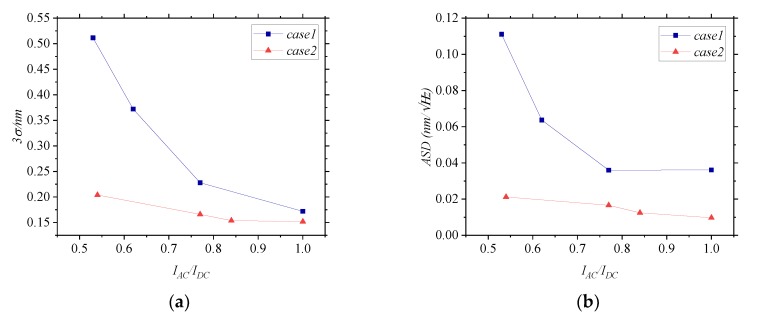
(**a**) The 3σ of the *DE* vary with *I_AC_/I_DC_* in different cases. (**b**) The ASD of the *DE* vary with *I_AC_/I_DC_* in different cases. In case 1, the interference angle is changed. In case 2, the irradiance ratio is changed.

As shown in Figure 8, the two factors that influence fringe contrast affect the error caused by disturbed MMFs differently. In case 1, the interference angle between the measurement arm and the reference arm changes, and in case 2, the ratio between the irradiance of the measurement arm and the irradiance of the reference arm changes. In Figure 8a, when *I_AC_/I_DC_* changes from 50% to 100%, the 3σ of the *DE* in case 1 changes from 0.52 nm to 0.17 nm, and the change rate gradually decreases. The 3σ of the *DE* in case 2 remains between 0.2 nm and 0.15 nm, and is under a small degree of influence. In Figure 8b, when *I_AC_/I_DC_* changes from 50% to 100%, the ASD of the *DE* changes from 0.11 nm to 0.04 nm in case 1, and the change rate gradually decreases. In case 2, the ASD of the *DE* remains between 0.02 nm and 0.009 nm, and is affected slightly. It can be concluded that the larger the interference angle, the larger the error caused by disturbed MMFs. The influence of the irradiance ratio on the disturbance error is small. Moreover, the greater the interference angle, the greater the rate of change of the 3σ and ASD of the *DE* with *I_AC_/I_DC_*. The reason for this can be that the greater the interference angle between the measurement arm and the reference arm, the greater the difference between the path *p*_m_ and *p*_r_ in Equation (9). However, when the interference angle is not changed, and only the irradiance of the beams is changed, *p*_m_ and *p*_r_ remain almost unchanged, essentially indicating that α(t) is a constant.

## 4. Conclusions

In this paper, the error caused by disturbed MMFs in the displacement measurement interferometer system with multi-mode fiber-coupled delivery is analyzed. When the MMF is disturbed, stress and deformation occur in it, which causes the ERI of the MMF changes. It is verified by simulation and experiment that, in the frequency domain, the error caused by disturbed MMFs is consistent with the frequency of disturbance, and the error has higher order frequency components. The effect of fringe contrast on the error caused by disturbed MMFs is studied experimentally. The ratio between the irradiance of the measurement arm and the irradiance of the reference arm and the interference angle between the measurement arm and the reference arm have the same effect on the electronic noise in the measurement results, but the effect on the error caused by disturbed MMFs is different. The irradiance ratio has less influence on the disturbance error, but the interference angle has greater influence on it. The larger the interference angle, the larger the disturbance error of the MMFs, and the larger the interference angle, the greater the rate of change of the measurement error with *I_AC_/I_DC_*.

The study focuses on an interferometer with multi-mode fiber-coupled delivery, and the MMFs are disturbed during the measurement. Because the perfect coincidence between the measurement arm and the reference arm cannot be realized, the error caused by disturbed MMFs cannot be avoided in the interferometer. It is worth mentioning that due to the influence of *I_AC_/I_DC_* on the error caused by disturbed MMFs, it is necessary to take the interference angle of the measurement arm and the reference arm as a design index when designing the displacement measurement interferometer system with multi-mode fiber-coupled delivery. There are some ways to make the measurement arm and the reference arm as close together as possible to achieve a tiny error, including by using flat convex lenses or retroreflectors. For interferometers without calibration, the disturbance amplitude and frequency are selected according to the operating conditions. Then, the interference angle range is determined by experiments to ensure that the errors caused by disturbed MMFs are within the allowable range. As shown in Figure 8, to ensure that the 3σ of the error is less than 200 pm and the ASD of the error is less than 40 pm, it is necessary to ensure that the interference angle is within a certain range so that *I_AC_/I_DC_* is above 90%.

## Figures and Tables

**Table 1:** The parameters in simulation.

Parameters	Value	Description
*λ*	633 nm	Free space wavelength
*n_1_*	1.3982	Refractive index of the cladding
*n_2_*	1.4584	Refractive index of the core
*d_1_*	425 μm	Outer diameter of the cladding
*d_1_*	400 μm	Diameter of the core
*L*	1 mm	Length of MMF
*E*	78 GPa	Young’s modulus
*ρ*	2203 kg/m^3^	Density
*μ*	0.17	Poisson’s ratio
*B_1_*	0.65 × 10^−12^ m^2^/N	First stress optical coefficient
*B_2_*	4.2 × 10^−12^ m^2^/N	Second stress optical coefficient

## References

[1] Knarren B.A., Cosijns S.J., Haitjema H., Schellekens P.H. (2005). Fiber characterization for application in heterodyne laser interferometry with nanometer uncertainty, part I: Polarization state measurements. Opt. Eng..

[2] Knarren B.A., Cosijns S.J., Haitjema H., Schellekens P.H. (2005). Fiber characterization for application in heterodyne laser interferometry, part II: Modeling and analysis. Opt. Eng..

[3] Ellis J.D., Meskers A.J., Spronck J.W., Schmidt R.H.M. (2011). Fiber-coupled displacement interferometry without periodic nonlinearity. Opt. Lett..

[4] Meskers A.J., Spronck J.W., Schmidt R.H.M. (2014). Validation of separated source frequency delivery for a fiber-coupled heterodyne displacement interferometer. Opt. Lett..

[5] Yang F., Zhang M., Ye W., Wang L. (2019). Three-degrees-of-freedom laser interferometer based on differential wavefront sensing with wide angular measurement range. Appl. Opt..

[6] Ye W., Zhang M., Zhu Y., Wang L., Hu J., Li X., Hu C. (2018). Translational displacement computational algorithm of the grating interferometer without geometric error for the wafer stage in a photolithography scanner. Opt. Express.

[7] Yang F., Zhang M., Zhu Y., Ye W., Wang L., Xia Y. (2019). Two Degree-of-Freedom Fiber-Coupled Heterodyne Grating Interferometer with Milli-Radian Operating Range of Rotation. Sensors.

[8] Agrawal G.P., Mumtaz S., Essiambre R.-J. Nonlinear performance of SDM systems designed with multimode or multicore fibers. Proceedings of the Optical Fiber Communication Conference.

[9] Mumtaz S., Essiambre R.-J., Agrawal G.P. (2012). Reduction of nonlinear penalties due to linear coupling in multicore optical fibers. IEEE Photonics Technol. Lett..

[10] San Fabián N., Socorro-Leránoz A.B., Del Villar I., Díaz S., Matías I.R. (2019). Multimode-coreless-multimode fiber-based sensors: Theoretical and experimental study. J. Lightwave Technol..

[11] Zhang S., Deng S., Wang Z., Yang W., Sun C., Chen X., Ma Y., Li Y., Geng T., Sun W. (2019). Optimization and experiment of a miniature multimode fiber induced-LPG refractometer. OSA Contin..

[12] Fermann M.E. (1998). Single-mode excitation of multimode fibers with ultrashort pulses. Opt. Lett..

[13] Gloge D., Marcatili E.A.J. (1973). Multimode theory of graded-core fibers. Bell Syst. Tech. J..

[14] Koplow J.P., Kliner D.A., Goldberg L. (2000). Single-mode operation of a coiled multimode fiber amplifier. Opt. Lett..

[15] Poletti F., Horak P. (2008). Description of ultrashort pulse propagation in multimode optical fibers. IOSA B.

[16] Hansen T.P., Broeng J., Libori S.E., Knudsen E., Bjarklev A., Jensen J.R., Simonsen H. (2001). Highly birefringent index-guiding photonic crystal fibers. IEEE Photonics Technol. Lett..

[17] Schriemer H.P., Cada M. (2004). Modal birefringence and power density distribution in strained buried-core square waveguides. IEEE J. Quantum Electron..

[18] Stone J. (1988). Stress-optic effects, birefringence, and reduction of birefringence by annealing in fiber Fabry-Perot interferometers. J. Lightwave Technol..

[19] Hsu C.-C., Chen H., Chiang C.-W., Chang Y.-W.J.O.e. (2017). Dual displacement resolution encoder by integrating single holographic grating sensor and heterodyne interferometry. Opt. Express.

[20] Jiang M.L., Li F.P., Wang X.D. (2011). Mini-nano-displacement measurement with double diffraction grating. Adv. Mater. Res..

[21] Lee C., Lee S.-K. (2013). Multi-degree-of-freedom motion error measurement in an ultraprecision machine using laser encoder. J. Mech. Sci. Technol..

[22] Lin C., Yan S., Ding D., Wang G. (2018). Two-dimensional diagonal-based heterodyne grating interferometer with enhanced signal-to-noise ratio and optical subdivision. Opt. Eng..

[23] Lu Z., Wei P., Wang C., Jing J., Tan J., Zhao X. (2016). Two-degree-of-freedom displacement measurement system based on double diffraction gratings. Meas. Sci. Technol..

[24] Wang X., Dong X., Guo J., Xie T. (2003). Two-dimensional displacement sensing using a cross diffraction grating scheme. J. Opt. A Pure Appl. Opt..

[25] Castenmiller T., van de Mast F., de Kort T., van de Vin C., de Wit M., Stegen R., van Cleef S. Towards ultimate optical lithography with NXT: 1950i dual stage immersion platform. Proceedings of the Optical Microlithography XXIII.

[26] Ellis J.D. (2014). Field Guide to Displacement Measuring Interferometry.

